# Transcutaneous Vagus Nerve Stimulation (tVNS) Enhances Response Selection During Sequential Action

**DOI:** 10.3389/fpsyg.2018.01159

**Published:** 2018-07-06

**Authors:** Bryant J. Jongkees, Maarten A. Immink, Alessandra Finisguerra, Lorenza S. Colzato

**Affiliations:** ^1^Cognitive Psychology Unit and Leiden Institute for Brain and Cognition, Leiden University, Leiden, Netherlands; ^2^School of Health Sciences and Cognitive Neuroscience Laboratory, University of South Australia, Adelaide, SA, Australia; ^3^Department of Cognitive Psychology, Institute of Cognitive Neuroscience, Faculty of Psychology, Ruhr University Bochum, Bochum, Germany; ^4^Institute for Sports and Sport Science, University of Kassel, Kassel, Germany

**Keywords:** tVNS, implicit motor sequence learning, response selection, GABA, cognitive enhancement

## Abstract

Transcutaneous vagus nerve stimulation (tVNS) is a non-invasive and safe technique that transiently enhances brain GABA and noradrenaline levels. Although tVNS has been used mainly to treat clinical disorders such as epilepsy, recent studies indicate it is also an effective tool to investigate and potentially enhance the neuromodulation of action control. Given the key roles of GABA and noradrenaline in neural plasticity and cortical excitability, we investigated whether tVNS, through a presumed increase in level of these neurotransmitters, modulates sequential behavior in terms of response selection and sequence learning components. To this end we assessed the effect of single-session tVNS in healthy young adults (*N* = 40) on performance on a serial reaction time task, using a single-blind, sham-controlled between-subject design. Active as compared to sham tVNS did not differ in terms of acquisition of an embedded response sequence and in terms of performance under randomized response schedules. However, active tVNS did enhance response selection processes. Specifically, the group receiving active tVNS did not exhibit inhibition of return during response reversals (i.e., when trial *N* requires the same response as trial *N*–2, e.g., 1-2-1) on trials with an embedded response sequence. This finding indicates that tVNS enhances response selection processes when selection demands are particularly high. More generally, these results add to converging evidence that tVNS enhances action control performance.

## Introduction

Non-invasive methods of brain stimulation have become an increasingly popular approach to probing the relationship between neurochemistry and cognitive-behavioral performance. Although transcranial direct current stimulation (tDCS) is currently the subject of great scientific interest (Plewnia et al., 2015), it has recently been suggested that transcutaneous (through the skin) vagus nerve stimulation (tVNS) may be a novel technique to investigate and potentially enhance the neuromodulation of action control (van Leusden et al., 2015). Converging evidence from animal and clinical studies suggests that tVNS increases levels of GABA (Ben-Menachem et al., 1995; Marrosu et al., 2003) and noradrenaline (NA) in the brain (Roosevelt et al., 2006; Raedt et al., 2011). Consistent with this literature, tVNS has been shown to increase intracortical inhibition in healthy adults (Capone et al., 2015), supporting the idea that tVNS might alter and potentially enhance performance related to the GABAergic and noradrenergic systems. Given the crucial role for GABA in the neuromodulation of response selection (Bar-Gad et al., 2003; Munakata et al., 2011; de la Vega et al., 2014) and motor learning (Floyer-Lea et al., 2006; Stagg et al., 2011), we investigated the effects of tVNS on implicit sequence learning and response selection processes underlying sequential action.

The neurochemical effects of tVNS have the potential to alter cortical excitability and synaptic plasticity, which are shaped by brain GABA concentration (Nakamura et al., 1997; Werhahn et al., 1999; Floyer-Lea et al., 2006; Boy et al., 2010; Stagg et al., 2011; Ziemann et al., 2015). Consistent with this neuromodulatory role, individual differences in GABA level have been related to response selection and inhibition (Snyder et al., 2010; Munakata et al., 2011; de la Vega et al., 2014), impulsivity (Boy et al., 2011), error detection, and conflict monitoring (van Veen and Carter, 2006), as well as implicit motor learning (Stagg et al., 2011; de Beaumont et al., 2012). The effects of GABA on response selection and inhibition are commonly explained by its role in a “winner-takes-all” mechanism, in which GABA enhances the mutual inhibition of competing response options (Bar-Gad et al., 2003; Plenz, 2003). This is thought to facilitate the suppression of incorrect response alternatives and aid in selection of the appropriate response (Munakata et al., 2011; de la Vega et al., 2014). Given this facilitatory effect of GABA on action control, it is possible that tVNS, via a transient increase in GABA concentration, modulates, and potentially enhances response selection processes (c.f. van Leusden et al., 2015).

Recent studies support this hypothesis by showing that tVNS can indeed improve cognitive-behavioral performance. These effects of tVNS were not related to sequenced action specifically, defined here as a sequence of movements that are serially ordered to achieve a task goal (Sakai et al., 2004; Abrahamse et al., 2013). However, previous work has demonstrated that tVNS can enhance processes thought to underlie motor sequence performance and learning. For example, Beste et al. (2016) demonstrated improved inhibitory control from tVNS. As robust response selection is crucial to sequenced actions (Deroost and Soetens, 2006), enhanced inhibition from tVNS might facilitate selection of the target response through suppression of competing non-target alternatives (Munakata et al., 2011; de la Vega et al., 2014; Colzato et al., 2018). Consistent with this notion, Steenbergen et al. (2015) reported that tVNS enhanced response selection when two responses were executed in succession.

Importantly, tVNS affects not only GABA but the NA system as well (Roosevelt et al., 2006; Raedt et al., 2011). In line with this finding, tVNS has been reported to enhance processes that (i) are associated with the acquisition of sequenced movements, and (ii) are thought to be mediated by NA transmission. For example, tVNS has been shown to enhance the formation of associative memory (Jacobs et al., 2015). When responses follow an implicit sequential structure, associative memory allows for development of an integrated representation of the sequence or sequence elements based on formed associations between responses (Hommel, 1996). Consistent with this notion, tVNS has been argued to improve associative memory via a presumed increase in NA transmission from the locus coeruleus to hippocampal areas (and the amygdala in the case of emotional memory formation) (Jacobs et al., 2015). Furthermore, increased post-error slowing is thought to be an important component of sequence learning (Ruitenberg et al., 2014) as it reflects upon rule-based performance (Tam et al., 2013). Sellaro et al. (2015) demonstrated that tVNS increased post-error slowing, which depends on catecholamine activity, i.e., dopamine (Moeller et al., 2012; Wardle et al., 2012) and NA (Ullsperger et al., 2010; Colzato et al., 2013). Taken together, the aforementioned findings support the hypothesis that tVNS can enhance response selection processes during sequential action.

In contrast to these expectations, there is also the possibility that tVNS results in suppression of sequential learning. Sequence acquisition is typically associated with an *increase* rather than a *decrease* in cortical excitability (Lin et al., 2011), and indeed, some have demonstrated that increased GABA predicts reduced implicit motor sequence learning (Stagg et al., 2011; de Beaumont et al., 2012). In light of these previous studies, the effect of tVNS on sequence acquisition remains uncertain. Therefore, the present study set out to clarify the effect of tVNS on sequence acquisition and response selection during sequential action.

### The Present Study

In more general terms, with the present study we set out to extend the literature on tVNS enhancement of cognitive-behavioral performance by investigating its potential to improve sequential action control. Given that tVNS increases brain GABA, which is crucial to the modulation of action control processes (Bar-Gad et al., 2003; Floyer-Lea et al., 2006; Munakata et al., 2011; Stagg et al., 2011; de la Vega et al., 2014), we tested the hypothesis that tVNS might enhance sequential action as assessed on a serial reaction time (SRT) task (Nissen and Bullemer, 1987). The SRT task is a 4-choice reaction time task that involves response selection, inhibition of non-target responses and implicit formation of response sequence structures, each of which may be sensitive to GABA and NA changes from tVNS. Typically, a second-order conditional (SOC) response sequence is embedded in the task unbeknownst to the participants. Implicit acquisition of the sequence structure results in increasingly shorter response latencies and less response errors as the task progresses (Nissen and Bullemer, 1987; Abrahamse and Noordzij, 2011; Schwarb and Schumacher, 2012). However, there is potential difficulty in disentangling the nature of these improvements (Jongkees et al., 2017a) as performance improvements might not necessarily be due to implicit learning processes but rather reflect general practice effects (Abrahamse and Noordzij, 2011). For this reason, a transfer approach is used to judge the extent by which performance improvements rely on the practiced sequence (Willingham, 1999; Robertson, 2007; Abrahamse and Noordzij, 2011). In the SRT task variation employed in the present experiment, each block of trials included both an embedded SOC sequence as well as a transfer sequence based on a pseudo-random stimulus presentation schedule. In addition to evaluating performance improvement across practice, this approach allowed for comparisons between sequenced trials and randomized trials as an index of sequence learning. Post-error slowing was also evaluated for trials under sequenced and random schedules to investigate the effects of tVNS on sequence learning processes. As tVNS might not enhance sequence learning but rather improve response selection processes, overall task accuracy and reaction time (RT) performance was assessed under the view that increased accuracy or reduced response latency under tVNS reflects efficiency of selecting the target response. To probe inhibitory processes that are relied upon to select target responses, we applied the concept of inhibition of return (Posner and Cohen, 1984; Lupiáñez et al., 1999 for reviews; see Klein, 2000) to the SRT paradigm to further investigate response selection processes under tVNS. In the SRT task, inhibition of return is evaluated by comparing RT on reversal trials to non-reversal trials (Vaquero et al., 2006). A reversal trial is defined as occurring when the target response location for trial *N* is a repetition of the target response location for trial *N-2* (e.g., 1-2-1; Vaquero et al., 2006). Longer response latencies for reversal trials as compared to non-reversal trials reflect inhibition of an action that has been recently performed (Klein, 2000). Increased GABA levels due to tVNS might result in suppression of inhibition of return as well as the inhibition of competing response options, thereby allowing efficient selection of a response even when it has been recently performed.

## Materials and Methods

### Participants

Forty undergraduate students from Leiden University were offered partial course credit for participation in a study on tVNS. Participants were randomly assigned to either the active (*N* = 20) or sham (*N* = 20) tVNS group. The groups were comparable with respect to age (*M* = 22.3 vs. 22.5 years, *SD* = 2.7 vs. 2.5, respectively), *t*(38) = 0.244, *p* = 0.809, and gender distribution, (*F:M* = 14:6 vs. 18:2, respectively), *X^2^*(1, *N* = 40) = 2.50, *p* = 0.114. Participants were screened individually using the Mini International Neuropsychiatric Interview (MINI), a short, structured interview of approximately 15 min that screens for several psychiatric disorders and drug use (Sheehan et al., 1998), and has been used previously in neuromodulation research (Jongkees et al., 2017a,b). Participants were included if they met the following criteria: (i) between 18 and 30 years; (ii) no history of neurological or psychiatric disorders; (iii) no history of substance abuse or dependence; (iv) no chronic or acute medication; and (v) no implants or cardiac disorders for safety reasons concerning the tVNS. Before the start of the study, participants were informed of the procedure and potential side-effects of the tVNS (i.e., itching, stinging, or burning sensation from the electrodes, reddening of the skin and head ache). None of the participants reported major side-effects. The study conformed to the ethical standards of the declaration of Helsinki with written informed consent from all subjects and the protocol was approved by the local ethical committee (Leiden University, Institute for Psychological Research).

### Transcutaneous Vagus Nerve Stimulation

**FIGURE 1:**
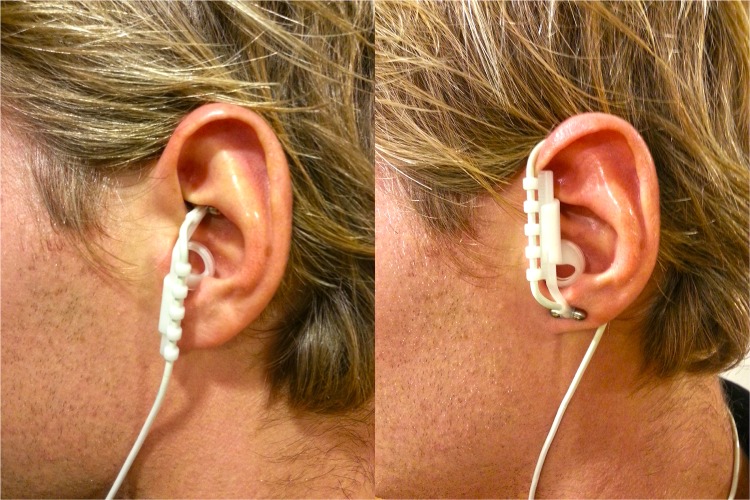
Positioning of the tVNS electrodes in the active (left) and in the sham (right) condition.

tVNS stimulates the afferent auricular branch of the vagus nerve, which is located medial of the tragus at the entry of the acoustic meatus (Kreuzer et al., 2012). In order to avoid stimulation of fibers to the heart, tVNS is safe to be applied to the left but not the right ear (Sperling et al., 2010; Kreuzer et al., 2012). The tVNS device consisted of two titan electrodes mounted on a gel frame and connected to a wired neurostimulating device (CMO2, Cerbomed, Erlangen, Germany), see **Figure 1**. Following the suggestions by Dietrich et al. (2008) for optimal stimulation, the tVNS^®^ device was programmed to a stimulation intensity of 0.5 mA, delivered with a pulse width of 200–300 μs at 25 Hz. Both active and sham stimulation constantly alternated between active stimulation for 30 s, followed by a break of 30 s. Consistent with Kraus et al. (2007), sham stimulation was applied by placing the electrodes over the center of the left ear lobe instead of the outer auditory canal, as the ear lobe is free of vagus innervation (Peuker and Filler, 2002) and its stimulation produces no activation in the cortex and brain stem (Kraus et al., 2013).

### Serial Reaction Time Task

To assess response selection and sequence learning, participants performed an adapted SRT task (Vaquero et al., 2006) presented using E-Prime 2.0 software (Psychology Software Tools, Inc., Pittsburgh, PA, United States). In this task four horizontally aligned empty squares are presented in the center of the screen. On each trial one of the squares turns red and the participant must press a corresponding button on the QWERTY keyboard (from left to right: V, B, N, M) using the index and middle fingers of the left (V, B) and right (N, M) hand. An error sound is presented if the wrong button is pressed, along with the Dutch words “Verkeerde toets!” (“Wrong button!”). RT is measured in milliseconds as the latency in the key press to the stimulus and if RT exceeds 3,000 ms, the Dutch words “Te langzaam!” (“Too slow!”) are presented. Following the response, the four empty squares appear for a 50 ms response-stimulus interval before the next stimulus is presented. Participants were instructed that accuracy and response speed were equally important in the task.

Participants completed 3 task familiarization blocks of 120 randomly sequenced trials prior to stimulation, and then performed 15 experimental blocks each consisting of 10 cycles of 12 trials while stimulation was applied. Each experimental block alternated between a cycle of random trials and two cycles of SOC trials (R-SOC-SOC-R-SOC-SOC-R-SOC-SOC-R), with each SOC cycle containing the same 12-item response sequence (VBVNMBNVMNBM) (Reed and Johnson, 1994). Whereas performance gradually improves on SOC trials as the response sequence is implicitly learned, the random response sequence prevents anticipation of responses and thus requires stimulus-oriented control. Hence RT and response errors are expected to be higher on random cycles (Willingham et al., 1989) but performance is expected to recover on SOC trials. After completion of each block, performance feedback indicated the number of errors and mean RT followed by a 30 s rest interval. The task took approximately 30 min to complete.

The random response sequences were generated prior to the study and held constant across all participants, to avoid chance-based group differences in the structure of the random cycles. For example, performance artifacts may occur due to differences in the number of reversal trials (Reed and Johnson, 1994; Vaquero et al., 2006). A reversal trial occurs when the third trial of any three consecutive trials involves the same target response as the first trial (e.g., V-B-V). Random cycles were generated to match SOC cycles on the number of reversals and hand switches (left-to-right and right-to-left) across trials (Jongkees et al., 2017a) and immediate response repetitions were not allowed within a random cycle nor at the transition between a random and SOC cycle. As such, any group difference in performance is not confounded by chance-based differences in the structure of random cycles.

### Procedure

Upon entering the lab, informed consent was obtained and participants practiced the SRT task to familiarize themselves with the task. Subsequently tVNS was applied and after 15 min of stimulation the experimental SRT task was started. Stimulation was applied throughout the entire task, which took on average 30 min. After the task participants were asked to rate, on a five-point (1–5) scale, to what extent they experienced (i) headache, (ii) neck pain, (iii) nausea, (iv) muscle contraction in the face and/or neck, (v) stinging sensation under the electrodes, (vi) burning sensation under the electrodes, (vii) uncomfortable (non-specific) feelings, and (viii) other sensations or adverse effects. None of the participants reported major side-effects.

### Statistical Analyses

The percentage of response accuracy (ACC) and mean RT for SRT task familiarization performance was calculated for each individual participant. RT calculation was based on correct trials only. ACC and RT for task familiarization were submitted separately to univariate analysis to test for any Group performance differences prior to stimulation conditions.

For performance in SRT task experimental blocks, ACC was calculated for each individual according to Sequence Type (SOC, random) and Trial Type (non-reversal, reversal) factors and submitted to a 2 (Group: active, sham) × 2 (Sequence Type: SOC, random) × 2 (Trial Type: non-reversal, reversal) analysis of variance (ANOVA) with repeated measures on the last two factors. RT was calculated based on correct trials according to Sequence Type, Trial Type and Block (1–15) factors. RT was then submitted to a 2 (Group) × 2 (Sequence Type) × 2 (Trial Type) × 15 (Block) ANOVA with repeated measures on the last three factors. For the purpose of the present experiment, a significant Group × Sequence Type × Block interaction was identified as being a critical test of enhanced sequence learning during active stimulation. A significant main effect of Group or a significant Group × Sequence Type interaction represented key identifiers of response selection efficacy. Enhanced response selection during active stimulation based on suppression of inhibition of return was expected to be revealed either as a significant Group × Trial Type interaction or a Group × Sequence Type × Trial Type interaction. Analysis for inspection of post-error slowing involved aggregating correct trial RT separately for post-error trials (a correct trial that was preceded by an error trial), post-correct trials (a correct trial succeeding a correct trial) under SOC and random sequence types. RT was then submitted to a 2 (Group) × 2 (Preceding Error) × 2 (Sequence Type) ANOVA with repeated measures on the last two factors. A significant Group × Preceding Error or Group × Preceding Error × Sequence Type interaction was identified as reflecting active and sham stimulation differences on post-error slowing.

Mauchly’s test was used to test the sphericity assumption for repeated measures ANOVA. Where sphericity was violated, a Huynh–Feldt correction was applied to the *p*-value. Significant interactions were further analyzed using Fisher’s LSD *post hoc* comparisons. For all analyses, a criterion of *p* < 0.05 was used to infer significant effects, interactions and differences.

## Results

Accuracy and RT performance during familiarization of the SRT task did not significantly differ between active and sham stimulation groups; *p* = 0.12 and *p* = 0.64, respectively. ACC performance was very high during experimental blocks (97%) and did not significantly differ between stimulation groups (*p* = 0.57), and there were no significant interactions between the Group factor and Sequence Type and Trial Type factors (*p*’s > 0.39).

**FIGURE 2:**
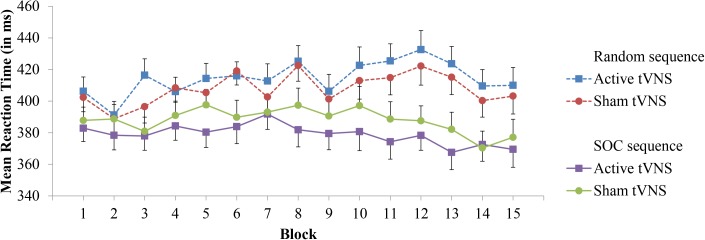
Mean reaction time in the SRT task as a function of block, sequence type, and tVNS group. Error bars reflect standard error of the means.

For experimental block RT performance, a significant Sequence Type × Block interaction (*F*[14,532] = 5.45, *p* < 0.0001, $\eta_{\text{p}}^{2}$ = 0.125) provides support for sequence learning within the SRT task, see **Figure 2**. With the exception of Block 2 (*p* = 0.19), RT was significantly lower on SOC sequence trials than random trials (*p’s* < 0.05). However, the Group × Block interaction (*p* = 0.89) was not significant. Important for the evaluation of sequence learning differences between stimulation groups, the Group × Sequence Type × Block interaction was not significant (*p* = 0.76). Further inspection of sequence learning based on assessment of post-error slowing did not reveal significant Group × Preceding Error (*p* = 0.27) or Group × Preceding Error × Sequence Type (*p* = 0.64) interactions, see **Table 1** for mean RTs. Thus, these results do not indicate that active tVNS stimulation enhanced sequence learning.

**Table 1:** Mean reaction time in ms as a function of stimulation group and trial type in the SRT task.

	Stimulation group	
Trial type	Sham	Active
Random order trials (overall)	392 (8)	398 (8)
Reversal trials	426 (9)	434 (9)
Non-reversal trials	389 (8)	395 (8)
Post-correct trials	387 (8)	395 (8)
Post-error trials	513 (18)	504 (18)
SOC trials (overall)	373 (9)	373 (9)
Reversal trials	406 (11)	386 (11)
Non-reversal trials	370 (9)	372 (9)
Post-correct trials	369 (9)	371 (9)
Post-error trials	511 (19)	489 (19)

*Standard error of the mean presented in parentheses. SOC, second order conditional sequenced trials.*

**FIGURE 3:**
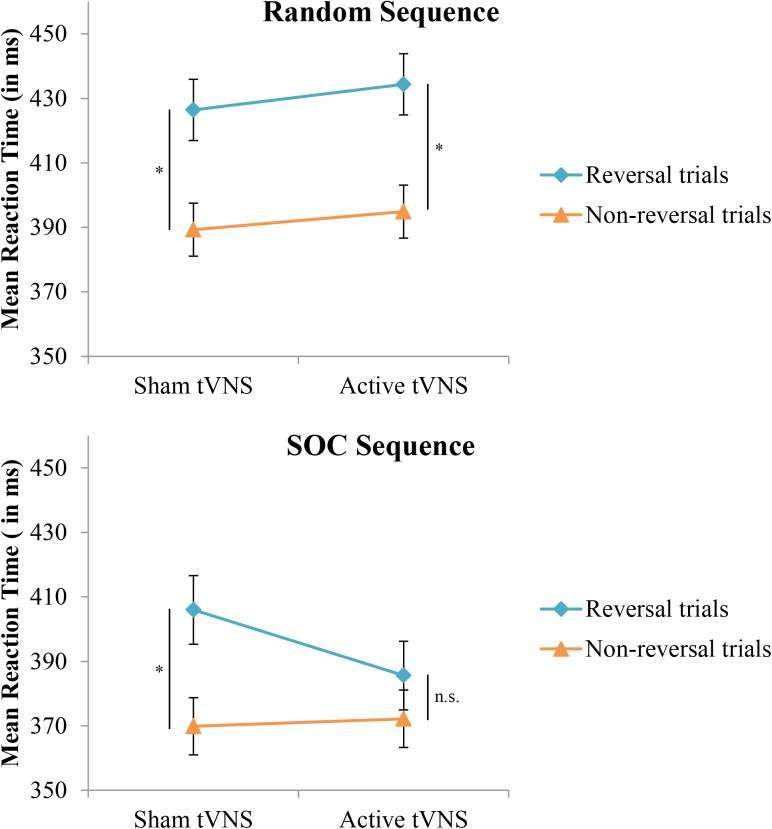
Mean reaction time in the SRT task as a function of trial type, sequence type, and tVNS group. Error bars reflect standard error of the means. Whereas both groups demonstrate a typical increase in reaction time on reversal trials during random response sequences, this increase is eliminated in the active tVNS group on trials with an embedded (SOC) response sequence. ^∗^*p* < 0.001.

With respect to the evaluation of response selection enhancement, neither the Group effect (*p* = 0.93) or the Group × Sequence Type interaction (*p* = 0.07) for RT were significant. In terms of inhibition of return as an index of response selection efficacy, the stimulation groups did not significantly differ between non-reversal trials and reversal trials (*p* = 0.16). However, a significant Group × Sequence Type × Trial type interaction (*F*[1,38] = 5.05, *p* < 0.05, $\eta_{\text{p}}^{2}$ = 0.117) indicated that enhancement of response selection through suppression of inhibition of return depended on the nature of the sequence structure that the reversal trial was performed in, see **Figure 3** and **Table 1**. Specifically, under active stimulation and in SOC sequence trials, RT was not significantly different between non-reversal (*M* = 372, *SE* = 9 ms) and reversal trials (*M* = 386, *SE* = 11 ms; *p* = 0.10). In contrast, under sham stimulation, RT for SOC sequence trials was significantly longer for reversal trials (*M* = 406, *SE* = 11 ms) than non-reversal trials (*M* = 370, *SE* = 9 ms; *p* < 0.0001). For random trials, both active and sham stimulation groups demonstrated significantly longer RT for reversal trials and non-reversal trials (both comparisons, *p* < 0.001). Nevertheless, under active stimulation, there were no significant differences between SOC sequence reversal trials and random sequence non-reversal trials (*p* = 0.42). In sum, these results indicate active tVNS eliminated inhibition of return during SOC sequenced response schedules.

## Discussion

The present study demonstrates that single-session tVNS improves response selection during sequential action. Whereas individuals tend to slow their responses when a response sequence contains an immediate reversal (e.g., 1-2-1 instead of 1-2-3) (Vaquero et al., 2006), this inhibition-of-return-like effect was eliminated under active tVNS while participants carried out an implicitly learned response sequence. The effect of tVNS was exclusive to response latency and did not extend to response errors, suggesting that the results are not attributable to a change in the speed-accuracy trade-off. This finding provides convergent evidence for the potential of tVNS to enhance action control in healthy adults.

In particular, this beneficial effect of tVNS on response selection is consistent with a wide range of studies demonstrating that increased GABA concentration facilitates action control (van Veen and Carter, 2006; Snyder et al., 2010; Boy et al., 2011; Munakata et al., 2011; de la Vega et al., 2014). Consistent with a winner-takes-all mechanism of response selection (Bar-Gad et al., 2003; Plenz, 2003), a higher GABA concentration promotes intracortical inhibition, leading to a suppression of incorrect response alternatives and thereby facilitating selection of the correct response (Munakata et al., 2011; de la Vega et al., 2014). It is conceivable, then, that a tVNS-induced increase in GABA is most beneficial when it is particularly challenging for the target response to successfully inhibit incorrect response alternatives.

This notion converges on the present results. In the SRT task the target response on reversal trials matches the response on trial N-2, which appears to be suppressed as evidenced by slower responses on reversal as compared to non-reversal trials (Vaquero et al., 2006). This possibly reflects the fact that on reversal trials it takes longer for the target response to successfully inhibit the incorrect response alternatives. In this case, a tVNS-induced enhancement of GABA could potentially aid in the inhibition of these response alternatives. The consequent facilitatory effect on selecting the target response accounts for the lack of RT slowing on reversal trials in SOC sequences.

The stimulation did not enhance or diminish implicit motor sequence learning. However, of note was the low rate of implicit learning in both groups. The task structure might have limited the opportunity to acquire the SOC sequence due to alternation of random and SOC response cycles within each block. Although this structure served to offer a more balanced inspection of performance on randomly sequenced versus SOC sequenced trials, the high prevalence of and frequent switching towards random response sequences might have interfered with participants’ ability to acquire the SOC sequence by predisposing them to a stimulus-based rather than a plan-based action control style (c.f. Tubau et al., 2007). A reduced tendency for plan-based control might have then limited the potential for implicit learning to be modulated by tVNS. Therefore, we recommend the null-finding regarding tVNS and motor sequence learning to be examined in future studies that employ a more classic SRT task in which experimental blocks are strongly dominated by SOC cycles.

Notwithstanding the observed null-findings for sequence learning, the fact that tVNS selectively enhanced performance when response selection demands were high, is of potential theoretical interest and is reminiscent of a previous finding that tVNS enhanced inhibitory control only when working memory was also involved (Beste et al., 2016). In the present study tVNS selectively enhanced response selection on reversal trials during SOC cycles. From a neurobiological perspective, it is plausible that GABA’s inhibitory effects on response selection have greatest behavioral impact, and are more sensitive to manipulation, during conditions of response conflict when several response alternatives are strongly activated and when it is particularly challenging for the target response to be selected, such as on reversal trials in the SRT task. This might also explain the lack of an effect of tVNS on the majority of SOC trials (i.e., non-reversal trials), as these trials might have led to insufficient competition between responses alternatives for a manipulation of GABA to be behaviorally detectable.

Before concluding, the present study gives rise to some theoretical questions that need to be addressed in follow-up studies: (i) Considering that the SRT task version used in this study had a one-to-one mapping of stimuli to responses, the results cannot disentangle whether tVNS affected stimulus-based versus response-based mechanisms (c.f. van Veen et al., 2001). That is, it is not clear whether tVNS on reversal trials facilitated in particular responding to the same stimulus as on trial *N-2*, or whether the same facilitation would be observed when not the stimulus but only the response was repeated. (ii) In a similar vein, repetition of the *N-2* trial has been associated with the backward inhibition effect, where response slowing on trial *N* is attributed to inhibition of the task-set required on trial *N-2* (Mayr and Keele, 2000). Considering that the present SRT task did not involve multiple task-sets, it remains to be determined whether the influence of tVNS on trial *N-2* repetition is exclusive to response activation or extends to task-set activation as well. (iii) It is also uncertain whether tVNS affected specifically inhibition of return versus more general biases that promote an alternating pattern of responses (e.g., Cho et al., 2002; Jones et al., 2002). As such, it is necessary for future studies to consider these issues, starting for example with mapping responses to multiple stimuli in order to disambiguate these effects.

Furthermore, it is important to acknowledge that aside from GABA also the noradrenergic system can be affected by tVNS (Roosevelt et al., 2006; Raedt et al., 2011). A shortcoming of the present study is that its behavioral findings cannot unequivocally distinguish between effects on these different neurotransmitter systems. Although tVNS did not affect the components of SRT task performance linked with NA activity—i.e., sequence learning as supported by associative memory formation (Jacobs et al., 2015) and post-error slowing (Sellaro et al., 2015)—we cannot definitively conclude that NA did not contribute to our results. As such, future studies should provide clarity on this issue by for example including physiological markers of GABAergic and noradrenergic activity in an attempt to relate baseline differences and changes in these markers to tVNS-induced changes in SRT task performance.

To conclude, the present study extends the previous literature on tVNS and action control performance by showing that tVNS enhanced response selection processes during sequential action.

## Author Contributions

BJ, MI, and LC designed the study. AF ran the study. MI analyzed the data. BJ wrote the first draft of the manuscript. MI, AF, and LC edited the manuscript.

## Conflict of Interest Statement

The authors declare that the research was conducted in the absence of any commercial or financial relationships that could be construed as a potential conflict of interest. The reviewer A-KS declared a past co-authorship with the author LC to the handling Editor.
